# The Burden of Streptococcus Pneumoniae‐Related Admissions in Pediatric Population: A Retrospective Cohort Study Between Years 2018–2023 From a Southern Italian Region

**DOI:** 10.1002/hsr2.72807

**Published:** 2026-07-15

**Authors:** Pamela Di Giovanni, Giuseppe Di Martino, Mariarosaria Petrangelo, Valeria Donatiello, Federica Vaccaro, Livia Tognaccini, Edoardo Trebbi, Teresa Aita, Ferdinando Romano, Tommaso Staniscia

**Affiliations:** ^1^ Department of Medicine and Ageing Sciences “G. d'Annunzio” University of Chieti‐Pescara Chieti Italy; ^2^ Unit of Epidemiology and Health Statistic Local Health Authority of Pescara Pescara Italy; ^3^ School of Hygiene and Preventive Medicine “G. d'Annunzio” University of Chieti‐Pescara Chieti Italy; ^4^ Department of Public Health and Infectious Diseases “La Sapienza” University of Rome Rome Italy; ^5^ Local Health Authority of Avezzano‐Sulmona‐L'Aquila L'Aquila Italy

**Keywords:** epidemiology, Italy, public health, Streptococcus pneumoniae

## Abstract

**Background:**

Pneumococcus are often found in the respiratory tract of healthy people, especially children, and are a common cause of infections such as otitis media, sinusitis, conjunctivitis and community‐acquired pneumonia. In other cases, the bacteria can cause more serious conditions known collectively as invasive pneumococcal disease (IPD), which include meningitis, bacteremia and osteomyelitis.

**Aims:**

The aim of the present study was to determine the burden and incidence of hospitalizations caused by S. pneumoniae in the pediatric population resident in Abruzzo region, Italy.

**Methods:**

This was a retrospective study. All admissions performed during the period 2018–2023 were extracted from the hospital discharge records. Hospitalization rates were directly standardized for age and gender, accordingly to the population of Abruzzo region.

**Results:**

The annual hospitalization rate for SP pneumonia during the study period showed a significant decrease between the years 2018–2021 with an APC of −14.6 (95% CI: −36.1–0.6), followed by a substantial increase across the period 2021–2023 with a significant APC of 74.2 (95% CI: 27.9–127.3). For meningitis, an increasing trend was observed between 2018 and 2020, with an AAPC of 6.1 (95% CI: 1.6–10.9), followed by a decline in 2021 and 2022. Bacteremia was stable during the study period. Conclusions: This study shows that pneumococcal disease has a significant impact on the pediatric population and on the health care system. These findings highlight the importance of improving vaccination of newborns and monitoring the circulation of pneumococcal serotypes.

## 1 Introduction

Infections caused by Streptococcus pneumoniae (SP) are responsible for a significant morbidity and mortality worldwide, especially among infants and the elderly [1].

It is estimated that approximately one million children die each year from SP‐related disease [2].

SP is often found in the respiratory tract of healthy patients, especially among infants, and are a common cause of infections such as otitis media, sinusitis, community‐acquired pneumonia (CAP) and, less frequently, conjunctivitis. In other cases, the bacteria can cause more severe conditions known as invasive pneumococcal disease (IPD), which represents the presence of bacteria in a normally sterile site. Clinically, it causes meningitis, bacteremia and osteomyelitis, with more severe outcomes [3].

Any condition in which SP is present in the blood, cerebrospinal fluid or other normally sterile body sites is defined as IPD [4].

SP produces several virulence factors that play a role in the disease process. Among these, the polysaccharide capsule is the most important, which is necessary for host invasion and disease. The bacteria synthesize over 100 types of capsular polysaccharides, of which 23 are cause for 80%–90% of IPD cases [5].

Pneumococcal polysaccharide vaccines (PPVs) and conjugate vaccines (PCVs) are the two types of vaccines currently in use to target multiple serotypes of SP. PPVs, which contain the purified capsular antigens, were first developed and marketed in the early 1980s and include the 23‐valent vaccine (PPV‐23), which is still in use in most European countries today [3].

However, PPV‐23 is not effective in children < 2 years of age. So, in order to improve vaccine efficacy in infants, several pneumococcal conjugate vaccines (PCVs), in which capsular polysaccharides are conjugated to a carrier protein, have been licensed, overcoming most of the limitations of PPVs [5].

The first PCV marketed in the United States (US) in the 2000s for the pediatric population was PCV‐7, followed by PCV‐13, which provided protection against a broader range of serotypes than PCV‐7. Since PCV‐13 was introduced, the prevalence of S. pneumoniae infections has decreased substantially. In fact, a review of the literature on the cumulative 20‐year effect of PCVs in children under 5 years of age in the US in 2021 showed that PCVs contributed to 91% reduction in the incidence of IPD between 1997 and 2019. There was also a 66–79% reduction in the annual number of hospitalizations for pneumonia in children under 5 years of age [6].

The pneumococcal serotypes that cause disease have continued to evolve due to natural pressures and in response to the use of vaccines. Therefore, the continued development of new PCVs is critical to increase coverage against emerging, clinically relevant serotypes to extend protection globally while maintaining suppression of serotypes included in previous PCVs [7].

Before the introduction of the first PCV in 2001, SP infection was estimated to cause over 108,000 deaths each year in the European Union (EU), accounting for around 2.2% of all deaths. It also caused 5.3% of deaths in infants under 5 years of age. Since the introduction of pneumococcal vaccines, the epidemiology of IPD and other related diseases has changed significantly, leading to a decrease in mortality rates across all age groups in Europe and Italy [3].

Vaccine recommendations differ between the countries of the EU in relation to which vaccines are available and which age category are targeted [8].

In Italy, pneumococcal vaccination is currently actively offered free of charge to newborns in their first year of life and to persons aged 65 years and older [9].

In addition, some specific health conditions allow free access to immunization regardless of age, such as chronic conditions (heart disease, chronic lung disease, diabetes mellitus, etc.), cerebrospinal fluid leaks, cochlear implants, hemoglobinopathies, immunodeficiencies, asplenia, oncological and hematological disorders and others [3].

In 2024, the 20‐valent polysaccharide conjugate vaccine (PCV‐20) was included in the national immunization schedule with the approval of the Italian National Vaccine Prevention Plan (PNPV 2023–2025). The PCV‐20 was licensed by the EMA in February 2022 and by the AIFA on 14 May 2022 for use in people aged 18 years and older [9].

Subsequently, several clinical trials were conducted to evaluate the safety and efficacy of PCV‐20 in the pediatric population, and safety and immune response were found to be comparable to PCV‐13. Therefore, on 27 April 2023, the FDA approved PCV‐20 for the prevention of IPD also for children aged 6 weeks to 17 years [9].

Nevertheless, 17,700 confirmed cases of IPD were notified in the European Union (EU) in 2022 with a crude notification rate of 5.1 cases per 100,000 inhabitants. Age‐specific rates were highest in infants under 1 year of age, with 13.4 confirmed cases per 100,000 inhabitants, and in adults aged 65 or over. Of the 7000 cases of IPD with a known outcome in Europe during 2022, 895 (12.8%) died. In particular, the case fatality rate was 3.9% in infants < 1 year of age and 3.6% in children aged 1–4 years [8].

Epidemiological data on IPD in Italy, updated to 2023, show a rate of 3.02 per 100,000 inhabitants, and the age groups with the highest incidence were children < 1 year and adults > 64 years. In 2023, the incidence of the disease in children aged < 1 year was 10.41 cases per 100,000 inhabitants, higher than in the pre‐pandemic period (6.50 cases per 100,000 inhabitants in 2019) [10].

In terms of mortality due to invasive pneumococcal disease (IPD) in Italy, 280 deaths were reported in 2023 out of 1783 cases. In particular, in the pediatric population, there were 3 deaths in children aged < 5 years and 2 deaths in children aged 5–9 years [10].

So, IPD continues to represent a significant economic burden on the healthcare system in Italy [11], leading to a significant healthcare resource consumption and direct medical costs [1].

The aim of the present study was to evaluate the burden and incidence of hospitalizations caused by SP in the pediatric population (< 15 years of age) from Abruzzo region, Italy, between the years 2018 and 2023 using Hospital Discharge Records (HDRs).

## 2 Materials and Methods

This is a retrospective study carried out in Abruzzo Region during years from 2018 to 2023 among the pediatric population (< 15 years of age).

Abruzzo is a region in southern Italy with a population of about 1.2 million, whose health services are organized into four local health authorities (LHAs). Each LHA was assigned to a specific province with at least one tertiary hospital (hub) and several secondary hospitals (spokes).

Data on hospitalizations were extracted from the HDRs of the Abruzzo region, considering all hospitalizations recorded from 2018 to 2023.

The HDRs include data on patient demographics, the diagnosis‐related group (DRG) code used to classify the admission, up to six diagnoses (one principal diagnosis and up to five comorbidities) and up to six possible procedures that occurred during the hospitalization. Diagnoses and procedures were coded according to the International Classification of Diseases, 9th Revision, Clinical Modification (ICD‐9‐CM), the National Center for Health Statistics (NCHS), and the Centers for Medicare and Medicaid Services External, Atlanta, GA, USA [12, 13].

To assess the burden of SP, all HDRs mentioning SP pneumonia, SP bacteremia and SP meningitis were included in the study and considered as specific hospital admissions caused by SP. We also considered admission related to bacterial infections due to unspecified microorganism, as reported in Table 1. In any case where the etiology was not specified, a certain percentage could be attributed to SP, according to the specific percentage reported in the literature. In accord to recent literature, the percentage attributable to SP could be 36% for unspecified pneumonia, 20% for unspecified bacteremia and 58% for unspecified meningitis, as reported in other paper from Italy [4].

**Table 1:** Diagnosis codes used to select hospital admissions for SP.

S.P. Hospital admissions	ICD‐9‐CM codes
Pneumococcal Specified Pneumonias	
Pneumococcal Pneumonia	481
Unspecified Pneumonias	
Bacterial pneumonia, unspecified	482.9
Bronchopneumonia, organism unspecified	485
Pneumonia, organism unspecified	486
Pneumonia due to Streptococcus, unspecified	482.30
Unspecified Pneumonias with concomitant pneumococcal infection	482.9; 485; 486; 482.30 plus
Pneumococcus infection in conditions classified elsewhere and of unspecified site	041.2
Pneumococcal Specified Meningitis	
Pneumococcal meningitis	320.1
Unspecified Meningitis	
Streptococcal meningitis	320.2
Meningitis due to Gram‐negative bacteria, not elsewhere classified	320.82
Meningitis due to unspecified bacterium	320.9
Meningitis, unspecified	322.9
Unspecified Meningitis with concomitant pneumococcal infection	320.2; 320.82; 320.9; 322.9 plus
Pneumococcus infection in conditions classified elsewhere and of unspecified site	041.2
Pneumococcal Specified Bacteraemia	
Pneumococcal septicemia	038.2
Unspecified Bacteraemia	
Streptococcal septicemia	038.0
Unspecified septicemia	038.9
Bacteraemia	790.7
Unspecified Bacteraemia with concomitant pneumococcal infection	038.0; 038.9; 790.7 Plus
Pneumococcus infection in conditions classified elsewhere and of unspecified site	041.2

### 2.1 Statistical Analysis

Qualitative variables were expressed as frequencies and percentages. Continuous variables were reported as median and interquartile range (IQR).

Annual admission rates were calculated as the ratio of the number of hospitalizations with a diagnosis of SP, using the corresponding attributable proportions calculated as described above [4], to the population of Abruzzo per 100,000 inhabitants for each year of the study period.

Then, hospitalization rates obtained were directly standardized for age and gender, according to the population of Abruzzo region in the first year of the study (2018).

The demographic structure (gender and age) of the population of Abruzzo region for each year from 2018 to 2023 was obtained from the website of the Italian National Institute of Statistics (ISTAT) [14].

The Joinpoint model (Joinpoint version 4.6.0.0, 2018) was performed to examine time trends of standardized rates and to estimate the average annual percentage change (AAPC) with a relative 95% confidence interval (95% CI). The AAPC should be considered as a summary measure of the trend over the time interval, calculated as a weighted average of the annual percentage changes (APC). It means that the AAPC is obtained by combining the APC in such a way that each APC value contributes proportionally to its significance or duration within the reference period. Specifically, each APC calculated for a specific year is multiplied by a weight that reflects, for example, the number of years included in the calculation. This approach allows for a more representative measure of the overall trend over time, reducing the influence of anomalous fluctuations and providing a more stable view of the general direction of the trend. The final model was obtained as linear segments, linked at the join points that represented the best‐fitting model of the observed data. For all tests, a two‐taled *p*‐value less than 0.05 was considered as statistically significant. The statistical analysis was performed with STATA software v23 (StataCorp LLC, College Station, TX, USA).

### 2.2 Ethics Statement

The study was conducted in conformity with the regulations on data management of the Regional Health Authority of Abruzzo and with the Italian Law on privacy (Art. 20‐21 DL 196/2003), published in the Official Journal, n. 190, on 14 August 2004. The data were encrypted prior to the analysis at the regional statistical office, when each patient was assigned a unique identifier. The identifiers eliminated the possibility of tracing the patients’ identities. According to Italian legislation, the use of administrative data does not require any written informed consent from patients.

## 3 Results

A total of 104,233 admissions from the Abruzzo region of Italy were examined during the study period (2018–2023).

The hospital admissions due to specific pneumococcal disease were 96 (0.09%), of which 87 (90.63%) cases were SP pneumonia, while considering IPD, 5 (5.21%) cases of bacteremia and 4 (4.17%) cases of meningitis occurred.

Instead, unspecified pathogens caused 2224 (2.13%) cases of pneumonia, 66 (0.06%) cases of bacteremia and 8 (0.01%) cases of meningitis. The percentage attributable to SP was calculated as described above and resulted in 800.64 (36%) cases of pneumonia, 13.2 (20%) cases of bacteremia and 4.64 (58%) cases of meningitis.

So, the majority of hospitalizations for SP were in children aged 1–4 years.

The most common type of discharge was “ordinary discharge at home” for a total of 76 (87.36%) children with SP pneumonia, while the remaining (11 cases, 12.64%) received other types of discharge.

Regarding SP meningitis, of the total 4 cases, 50% (2 cases) occurred in children aged 1 year and the other 50% (2 cases) in the 5–14 year age group. There were no cases of SP meningitis in the 1–4‐year age group, in contrast to SP pneumonia, where this age group was most affected.

SP meningitis had the longest length of stay (LOS) at around 12 (8.5–15.5) days, much longer than hospitalizations for SP pneumonia or SP bacteremia, which required LOS of 6 (5–8) and 9 (7–10) days, respectively.

No child hospitalized for pneumococcal disease required the Neonatal Intensive Care Unit (NICU). The characteristics of young patients with SP pneumonia, SP bacteremia and SP meningitis are described in more detail in Table 2.

**Table 2:** Observed hospital admissions from the Abruzzo region during 2018 to 2023 grouped by SP pneumonia, SP bacteremia and SP meningitis.

	Pneumonia		Bacteremia		Meningitis	
	*n* = 87		*n* = 5		*n* = 4	
Gender *n (%)*	N°	%	N°	%	N°	%
Male	46	52.87	2	40	1	25
Female	41	47.13	3	60	3	75
Group of Age *n (%)*						
< 1	5	5.75	3	60	2	50
1–4	60	68.96	1	20	0	0
5–14	22	25.29	1	20	2	50
Type of Discharge *n (%)*						
At Home	76	87.36	3	60	2	50
Others	11	12.64	2	40	2	50
Neonatal Intensive Care Unit (NICU) *n (%)*	0	0	0	0	0	0
Length of Stay *median (IQR)*	6 (5–8)		9 (7–10)		12 (8.5–15.5)	

The analysis of hospital admissions for non‐specific pneumonia during the study period showed that once again the most affected age group was 1–4 years with a total of 1310 (58.9%) cases. On the other hand, the lowest number of cases was recorded in the age group under 1 year with 316 (14.21%), while in the age group 5–14 years, there were 598 (26.89%) cases. Only three young (0.13%) patients required the Neonatal Intensive Care Unit (NICU).

As observed in SP bacteremia, the most affected children with unspecified bacteremia were those under 1 year of age, with a total of 29 cases (43.95%), followed by 17 cases (25.75%) in the age groups 1–4 years and 20 cases (30.3%) in the group 5–14 years.

The 6.06% of these patients needed for NICU.

Again, most young patients received “ordinary discharged at home” (83.33%) and only one patient died (1.51%); the remaining had others type of discharge (15.15%).

For unspecific meningitis, there were the same number of cases (3; 37.5%) in both the 5–14 age group and in children under 1 year of age. There were only two cases (25%) in the 1–4‐year age group. No child hospitalized with unspecified meningitis required Neonatal Intensive Care Unit (NICU).

Out of a total of 8 cases of unspecific meningitis, 5 (62.5%) received “ordinary discharge at home,” and 3 (37.5%) had other types of discharge. No patients died for unspecified meningitis.

In contrast to SP‐specific meningitis, unspecific meningitis had a shorter length of stay (LOS) of 2 (0–18) days. While unspecific pneumonia and unspecified bacteremia required a similar LOS as specific infection of SP, amounting to 5 (4–6) and 8 (5–12) days, respectively.

Table 3 details the characteristics of young patients with unspecified pneumonia, unspecified bacteremia and unspecified meningitis.

**Table 3:** Observed hospital admissions from the Abruzzo region during 2018 to 2023 grouped by unspecified pneumonia, unspecified bacteremia and unspecified meningitis.

	Unspec pneumonia		Unspec bacteremia		Unspec meningitis	
	*n* = 2224		*n* = 66		*n* = 8	
Gender	N°	%	N°	%	N°	%
Male	1208	54.32	42	63.63	5	62.5
Female	1016	45.68	24	36.36	3	37.5
Group of Age						
< 1	316	14.21	29	43.95	3	37.5
1 ≤ x < 5	1310	58.9	17	25.75	2	25
5 ≤ x ≤ 14	598	26.89	20	30.3	3	37.5
Type of Discharge						
Deaths	1	0.04	1	1.51	0	0
At Home	2030	91.28	55	83.33	5	62.5
Others	193	8.68	10	15.15	3	37.5
Neonatal Intensive Care Unit (NICU)	3	0.13	4	6.06	0	0
Length of Stay Median (IQR)	5 (4–6)		8 (5–12)		2 (0–18)	

The annual hospitalization rate for SP pneumonia during the study period showed a significant decrease between the years 2018–2021 with an APC of −14.6 (95% CI: −36.1–0.6), followed by a substantial increase across the period 2021–2023 with a significant APC of 74.2 (95% CI: 27.9–127.3), as reported in Table 4.

**Table 4:** Standardized hospitalization rate per 100,000 inhabitants due to pneumococcal infection in Abruzzo region (2018–2023).

Streptococcus pneumoniae admission rate+			
	Pneumonia	Bacteremia	Meningitis
2018	11.62	0	0.96
2019	12.82	0	0.97
2020	7.92	0.82	1.08
2021	7.15	0.83	0
2022	17.32	0.84	0
2023	20.98	1.62	1.01
AAPC	13.6 (2.1–24.5)	20.9 (−11.8–61.3)	6.1 (1.6–10.9)*

^+^ Admission rate was based on observed plus estimated cases.

* Trend in Meningitis was estimated till year 2020, while trend in Bacteremia was estimated from 2020 to 2023.

The hospitalization rate for SP bacteremia was stable during the study period, ranging from 0.80 per 100,000 in 2020 to 1.62 per 100,000 in 2023 [AAPC: 20.9 (95% CI: −11.8–61.3)].

For the hospital admission rate for SP meningitis, an increasing trend was observed between 2018 and 2020, with an AAPC of 6.1 (95% CI: 1.6–10.9), followed by a decline in 2021 and 2022, with no cases in these years, and then returned to levels similar to 2020 in year 2023.

When analyzing the differences between the trends in hospitalization rates for pneumococcal pneumonia in the different age groups studied, it was found that children aged 1–4 years were significantly more affected than the other two groups, with an AAPC of 16.1 (95% CI: 2.0–29.1). Specifically, the rate decreased from 2018 to 2021 with an APC of −8.6 (95% CI: −36.3–13.9) and then increased significantly from 2021 to 2023 with an APC of 66.2 (95% CI: 15.4–126.2).

The 5–14 age group, not the most affected by pneumococcal pneumonia, showed a similar trend to the 1–4 age group. From 2018 to 2020, there was a decrease with an APC of −47.2 (95% CI: −68.5–14.3) and from 2020 to 2023, there was an increase with an APC of 54.6 (95% CI: −3.2–211.5).

While for the under‐1 age group, the trend remained stable during the period investigated by the study (2018–2023) with an AAPC of 47.8 (95% CI: −99.9–373028).

As shown previously, males are more often hospitalized for pneumococcal pneumonia than females. In fact, when analyzing the differences in trend between the two genders, an AAPC of 0.3 (95% CI: −6.1–8.3) was found for males, while an AAPC of 33.6 (−3.3–84.4) was found for females.

In more detail, the trend analysis showed significant differences between males and females in specific year ranges over the entire study period. In particular, among males, there was a decrease in hospitalization rates for SP pneumonia from 2018 to 2020 with an APC of −37.1 (95% CI: −47.3 to −20.80), and then an increase from 2020 to 2023 with an APC of 37 (95% CI: 21.3–71.2).

## 4 Discussion

In the context of this retrospective study conducted in Abruzzo, a southern Italian Region, from 2018 to 2023, it was observed that pneumococcal disease has a significant burden in the pediatric population. This result was obtained by analyzing all HDRs of children residing in Abruzzo.

There was no uniform age group affected for all types of SP infection, but these varied according to the type of disease. For example, pneumococcal pneumonia most commonly affected children aged 1–4 years with a hospitalization rate of 24.08 cases per 100,000 inhabitants in 2023 (the highest number of cases was in the 1–4‐year age), while SP bacteraemia mainly affected children younger than 1 year (1.41 cases per 100,000 inhabitants in 2023), as reported in Table S1. On the other hand, pneumococcal meningitis was less common in children aged 1–4 years, whereas it was more evenly distributed in the extreme age groups.

The rate of IPD hospitalization in this study matched Italian national data on surveillance of invasive bacterial diseases. In 2023, the most at‐risk age group for IPD was children under 1 year old, with 10.41 cases per 100,000 [10].

So, children under 1 year of age were the most affected by invasive pneumococcal disease (IPD).

These data are also in line with those published in the Annual Epidemiological Report of the European Centre for Disease Prevention and Control (ECDC), which showed a rate of 13.4 per 100,000 in children under 1 year of age during 2022 [8].

The estimated hospitalization rate for SP pneumonia in children living in Abruzzo during the study period (2018–2023), showed a higher rate in males than in females. These were also found in other studies, such as that by Ruth Gil‐Prieto et al., who analyzed the burden of SP disease in Spain during 2016–2020 and observed a higher hospitalization rate in males than in females in all age groups, reaching statistically significant differences in those aged < 1 year [15].

However, the many changes in vaccination over the last two decades and their impact on pneumococcal hospitalization rates have made comparisons between countries difficult due to the different time periods of the studies [15].

The decrease in the trend of the hospitalization rate for PC pneumonia in children under 5 years of age can be explained by several external factors, such as the pandemic and vaccination rate. Data reported during the COVID‐19 pandemic in the Abruzzo region agrees with data published in the recent literature across Italy and Europe.

In fact, the hospitalization rate of the present study in this group ranged from 16.89 per 100,000 inhabitants in 2019 to 10.21 per 100,000 inhabitants in 2020, as reported in Table S1, like numerous other studies conducted in Italy and worldwide, in particular one by Principi et al. [16].

Typically, viral respiratory infections are associated with an increased risk of subsequent bacterial infections, particularly invasive disease and pneumonia. Therefore, there was a potential for increased rates of invasive bacterial disease following SARS‐CoV‐2 infection. Instead, government containment measures in response to COVID‐19 circulation and subsequent changes in human movement reduced respiratory viral and bacterial transmission. This could lead to a decline in invasive disease due to a concomitant reduction in transmission of respiratory‐associated bacteria [17].

The increase in incidence after the COVID‐19 pandemic, probably due to low exposure to SP as a result of the implementation of non‐pharmaceutical interventions (NPIs), may have led to a significant immunity debt in relevant population groups, favoring the development of infection [16].

Moreover, the COVID‐19 pandemic had a major negative impact on the population and on national health services, including many infectious diseases [12] [18], chronic degenerative diseases [19] and vaccination coverage. In particular, public health measures that forced people to stay at home led some persons to postpone planned vaccinations for themselves or their children. In addition, the need to reorganize health services to increase the availability of dedicated staff to respond to the emergency had an impact on routine vaccination activities, as evidenced by the decline in vaccination coverage in 2020 [20].

Vaccination coverage is one of the most important indicators for monitoring the results of the vaccination strategy and its implementation [20].

Data are collected and published annually by the Ministry of Health and are available online on the site “EpiCentro” [21].

Analysis of pneumococcal vaccination coverage among the 2021 birth cohort in the Abruzzo region showed that coverage at 24 months was 92.93% (updated to June 2024), below the 95% cut off recommended by the World Health Organization (WHO) [21]. However, the vaccination coverages increased after the pandemic period, when reached 88.53% in 2020.

Despite widespread vaccination, SP remains a major cause of IPD in the pediatric population and in the community. Surveillance of IPD is therefore essential to assess vaccine effectiveness, monitor the spread of resistant strains and identify emerging serotypes [22].

A study conducted by the Piedmont Region to assess the impact of anti‐pneumococcal vaccination between 2008 and 2022 concluded that there was an increased risk of infection with the S. pneumoniae Penicillin G resistance (PG‐R) strain in the infant population after the introduction of the vaccination policy. They also found that the development of bacteremia was more frequent in infants and children than in adults and the elderly. So, PCV vaccination of children may reduce the incidence of antimicrobial resistance (AMR) in the adult population [22].

A study conducted in the Veneto region of Italy assessed the economic impact of pneumococcal disease in children under 15 years of age following the introduction of PCV‐13 from 2010 to 2017. The results showed that replacing PCV‐7 with PCV‐13 had a significant impact on reducing the economic burden of pneumococcal disease, ranging from a total annual regional expenditure of approximately €8.88 million for SP pneumonia to €3.59 million. The same was true for the total annual expenditure attributed to IPD and syndromic invasive disease, which ranged from approximately €1.35 million to €1.02 million [23].

After the European Commission (EC) approved the use of PCV‐20 in the pediatric population on 12 March 2024, Germany conducted a cost‐effectiveness analysis comparing 20‐valent conjugate vaccines (PCV‐20) on a 3 + 1 schedule with PCV‐15 and PCV‐13, both on a 2 + 1 schedule. They found that PCV‐20 was the dominant vaccination strategy over the two lower‐valent alternative vaccines and was estimated to have a greater health impact, preventing more cases of pneumococcal disease over a 10‐year period. So, PCV‐20 has the potential to significantly reduce the clinical and economic burden of SP disease by offering much broader protection than the lower‐valent vaccines [24].

The main strength of this analysis is that the results obtained are based on real data extracted from HDRs, which are electronically recorded in official health databases of the entire population of the Abruzzo Region. In addition, a large number of HDRs (104,233) and a long study period (2018–2023) were evaluated, allowing the generalization of the results to the entire pediatric population. The impact of pneumococcal disease on this group of young people of the Abruzzo region can be used to assess possible preventive measures to increase vaccination compliance.

Another strength is the inclusion of multiple pneumococcal disease manifestations, both SP‐specific and those where SP is considered a potential causative agent, considering the attributable proportion calculated as described above [4].

A limitation of this study is the underestimation of total pneumococcal disease because not all cases are laboratory confirmed, and a blood culture may not be obtained or the patient may have already been treated with antibiotics, resulting in false negatives [25].

This adds to the uncertainty about the etiology of the conditions requiring hospitalization; indeed, it is not easy to estimate the morbidity of SP disease due to the questionable accuracy in compiling HDRs and due to possible errors in the assignment of diagnostic codes using ICD‐9‐CM [26]. In addition, this study was based only on HDRs, so out‐of‐hospital infections cannot be considered. However, the attributable fraction used in this study could overestimate the real incidence of the disease, particularly for SP meningitis. Finally, trend analyses in small subgroups and rare outcomes (e.g., bacteremia, meningitis, < 1 year) are based on very small numbers and should be interpreted with caution.

In conclusion, hospital admissions for SP still represent a heavy burden for healthcare services despite the significant reduction in morbidity and over the past 20 years.

The additional serotypes included in PCV‐20 are medically significant, so it would be advantageous to include higher‐valence PCVs, particularly PCV‐20, to protect people against them, considering that pneumococcal disease (PD) is one of the most common vaccine‐preventable diseases worldwide [27].

So, prevention is still the most precious tool for reducing the burden of SP disease, and vaccination strategies can be used for primary prevention to minimize the impact of SP in the population.

## 5 Conclusions

This study shows that pneumococcal disease has a significant impact on the pediatric population (under 15 years of age) and on the health care system of Abruzzo region. These findings highlight the importance of improving vaccination of new‐borns and monitoring the circulation of pneumococcal serotypes that cause pneumonia and invasive disease to optimize coverage with available vaccines and to reduce preventable hospitalizations.

## Author Contributions

**Pamela Di Giovanni:** conceptualization, writing – original draft, methodology, validation. **Giuseppe Di Martino:** conceptualization, methodology, formal analysis, writing – original draft. **Mariarosaria Petrangelo:** software, data curation, writing – review and editing. **Valeria Donatiello:** software, data curation, writing – review and editing. **Federica Vaccaro:** validation, writing – review and editing, visualization. **Livia Tognaccini:** validation, resources, writing – review and editing. **Edoardo Trebbi:** validation, investigation, writing – review and editing. **Teresa Aita:** investigation, visualization, writing – review and editing. **Ferdinando Romano:** visualization, writing – review and editing, supervision. **Tommaso Staniscia:** conceptualization, project administration, supervision.

## Funding

The authors have nothing to report.

## Conflicts of Interest

The authors declare no conflicts of interest.

## Supporting information


**Table S1:** hospitalization rate by age classes.

## Data Availability

The authors have nothing to report.
