# Supplementary material for: The Burden of Streptococcus Pneumoniae‐Related Admissions in Pediatric Population: A Retrospective Cohort Study Between Years 2018–2023 From a Southern Italian Region
Source: Health Sci Rep. 2026 Jul 15;9(7):e72807. doi: 10.1002/hsr2.72807 (PMC13373523; doi:10.1002/hsr2.72807)
Supplement: Supplementary file 1 — Table S1: hospitalization rate by age classes. [file HSR2-9-e72807-s001.docx]

|  |  | Pneumonia |  | Bacteremia |  | | Meningitis | | Annual rate by age group | Total annual rate |  |  |  |  |
| --- | --- | --- | --- | --- | --- | --- | --- | --- | --- | --- | --- | --- | --- | --- |
|  |  |  |  |  | |  | |  |  |  |  |  | |  |
| 2018 | <1 | 0 |  | 0 | | 1.58 | |  | 1.58 |  |  |  |  | |
|  | 1-5 | 10.88 |  | 0 | | 0 | |  | 10.88 |  |  |  |  | |
|  | 5-14 | 8.16 |  | 0 | | 0 | |  | 8.16 |  |  |  |  | |
|  | Total |  |  |  | |  | |  |  | 20.62 |  |  |  | |
| 2019 | <1 | 2.93 |  | 0 | | 0 | |  | 2.93 |  |  |  |  | |
|  | 1-5 | 13.96 |  | 0 | | 0 | |  | 13.96 |  |  |  |  | |
|  | 5-14 | 4.11 |  | 0 | | 1.59 | |  | 5.7 |  |  |  |  | |
|  | Total |  |  |  | |  | |  |  | 22.59 |  |  |  | |
| 2020 | <1 | 1.53 |  | 1.35 | | 1.77 | |  | 4.65 |  |  |  |  | |
|  | 1-5 | 8.68 |  | 0 | | 0 | |  | 8.68 |  |  |  |  | |
|  | 5-14 | 2.77 |  | 0 | | 0 | |  | 2.77 |  |  |  |  | |
|  | Total |  |  |  | |  | |  |  | 16.1 |  |  |  | |
| 2021 | <1 | 0 |  | 1.37 | | 0 | |  | 1.37 |  |  |  |  | |
|  | 1-5 | 8.92 |  | 0 | | 0 | |  | 8.92 |  |  |  |  | |
|  | 5-14 | 2.79 |  | 0 | | 0 | |  | 2.79 |  |  |  |  | |
|  | Total |  |  |  | |  | |  |  | 13.08 |  |  |  | |
| 2022 | <1 | 0 |  | 0 | | 0 | |  | 0 |  |  |  |  | |
|  | 1-5 | 17.14 |  | 1.37 | | 0 | |  | 18.51 |  |  |  |  | |
|  | 5-14 | 11.24 |  | 0 | | 0 | |  | 11.24 |  |  |  |  | |
|  | Total |  |  |  | |  | |  |  | 29.75 |  |  |  | |
| 2023 | <1 | 3.19 |  | 1.41 | | 0 | |  | 4.6 |  |  |  |  | |
|  | 1-5 | 24.08 |  | 0 | | 0 | |  | 24.08 |  |  |  |  | |
|  | 5-14 | 7.12 |  | 1.26 | | 1.65 | |  | 10.03 |  |  |  |  | |
|  | Total |  |  |  | |  | |  |  | 38.71 |  |  |  | |
| AAPC | <1 | 47.8(-99.9-373.0) | |  | |  | |  |  |  |  |  |  | |
|  | 1-5 | 16.1(2.0-29.1) |  |  | |  | |  |  |  |  |  |  | |
|  | 5-14 | 3.9(-17-34) |  |  | |  | |  |  |  |  |  |  | |

**Table S1.** **Hospitalization Rate by age classes**
